# Unraveling the Role of the microRNA-Mediated Regulation of Actin-Binding Proteins in Ovarian Cancer: A Narrative Review

**DOI:** 10.3390/cancers17142315

**Published:** 2025-07-11

**Authors:** Efthalia Moustakli, Anastasios Potiris, Athanasios Zikopoulos, Apostolia Galani, Konstantinos Kechagias, Grigorios Karampas, Ismini Anagnostaki, Chrysi Christodoulaki, Angeliki Gerede, Panagiotis Christopoulos, Nikolaos Thomakos, Sofoklis Stavros

**Affiliations:** 1 Laboratory of Medical Genetics, Faculty of Medicine, School of Health Sciences, University of Ioannina, 451 10 Ioannina, Greece; ef.moustakli@uoi.gr; 2 Third Department of Obstetrics and Gynecology, University General Hospital “ATTIKON”, Medical School, National and Kapodistrian University of Athens, 124 62 Athens, Greece; apotiris@med.uoa.gr (A.P.); thanzik92@gmail.com (A.Z.); isanagnostaki3@gmail.com (I.A.); christodoulakichr@hotmail.com (C.C.); 3 Department of Metabolism, Digestion and Reproduction, Faculty of Medicine, Imperial College London, London W12 ONN, UK; l.galani22@imperial.ac.uk (A.G.); konstantinos.kechagias18@imperial.ac.uk (K.K.); 4 Second Department of Obstetrics and Gynecology, University Hospital “Aretaieion”, Medical School, National and Kapodistrian University of Athens, 115 28 Athens, Greece; karampasgr@yahoo.gr (G.K.); panchrist@med.uoa.gr (P.C.); 5 Department of Obstetrics and Gynecology, Democritus University of Thrace, 691 00 Alexandroupolis, Greece; agerede@otenet.gr; 6 First Department of Obstetrics and Gynecology, Alexandra Hospital, Medical School, National and Kapodistrian University of Athens, 115 28 Athens, Greece; nthomakos@med.uoa.gr

**Keywords:** microRNA (miRNA), actin-binding proteins, ovarian cancer, cytoskeleton, biomarker, gene regulation

## Abstract

Ovarian cancer remains one of the most lethal gynecological malignancies, primarily due to its late diagnosis and limited prospects for successful treatment. MiRNAs have been shown to be important post-transcriptional regulators in a variety of cancer-related pathways in recent years. One of the principal mechanisms underlying the motility, invasiveness, and metastatic potential of ovarian cancer cells is the microRNA-mediated regulation of ABPs. As integral components of the cytoskeletal network, ABPs participate in dynamic cellular processes such as migration, adhesion, and invasion, and are critically involved in tumor development and progression. Recent data indicate that some miRNAs affect ABP expression and activity, which in turn affects cytoskeletal remodeling and, ultimately, tumor cell behavior. The role of miRNAs in cancer development is inherently complex due to their ability to function as both tumor suppressors and oncogenes, depending on the molecular context. Key ABPs that are targeted by particular miRNAs are discussed in terms of their clinical relevance, including their potential utility as diagnostic biomarkers or therapeutic targets. A deeper understanding of these regulatory pathways may offer new opportunities for early detection and personalized treatment strategies. In this narrative review, the current knowledge of how miRNAs affect ABP expression and function, and how this interaction contributes to the development and progression of ovarian cancer, is compiled.

## Simple Summary

Ovarian cancer remains one of the most lethal gynecological malignancies, and microRNAs (miRNAs) have been shown to be important post-transcriptional regulators in a variety of cancer-related pathways. MiRNA regulation of actin-binding proteins (ABPs) constitutes a key mechanism underlying the motility, invasiveness, and metastatic capacity of ovarian cancer cells. This narrative review aims to give a summary of what is currently known about the roles that ABPs play in ovarian cancer. Furthermore, this review aims to provide new insights and highlight potential intervention techniques by shedding light on the miRNA-mediated regulation of ABPs in the biology of ovarian cancer. MiRNAs regulate key tumorigenic processes such as cell migration, invasion, epithelial-to-mesenchymal transition, and metastasis by modulating the expression and function of vital cytoskeletal regulators. The dual roles of miRNAs as tumor suppressors and oncogenes are increasingly recognized, highlighting their complexity and therapeutic potential.

## 1. Introduction

The fifth most common cause of cancer-related mortality for women worldwide is ovarian cancer, a diverse and severe gynecologic disease. The overall five-year survival rate for ovarian cancer is still around 45%, despite notable advancements in platinum-based chemotherapy and cytoreductive surgery [1]. According to Sonkin and Thomas’ thorough analysis of cancer treatments from the past, present, and future, systemic issues like late-stage diagnosis, rapid metastasis, and the development of chemoresistance are the cause of this poor prognosis [2]. Therefore, the demand for more effective targeted therapies and early diagnostic biomarkers has become increasingly urgent [3].

Non-coding RNAs, of which miRNAs are the most well-known, have been identified in the last 20 years as essential post-transcriptional regulators of gene expression that operate in both healthy and diseased settings, including cancer [4]. Through binding to complementary sequences in the 3′ untranslated regions (UTRs) of target messenger RNAs (mRNAs), miRNAs, typically 20–24 nucleotides in length, regulate gene expression by inducing translational repression or mRNA degradation. Aberrant expression of miRNAs has been associated with the development, progression, and metastatic spread of multiple cancers, including ovarian cancer [5]. miRNAs have the capacity to function as tumor suppressors or oncogenes (oncomiRs), depending on their target genes and the cellular environment, thereby influencing critical cellular processes such as proliferation, apoptosis, angiogenesis, immune evasion, and resistance to therapy [6].

ABPs, one of the many downstream targets of miRNAs, primarily regulate the cytoskeleton, a dynamic structural framework that governs cell shape, polarity, migration, and division [7]. The processes of invasion, metastasis, and EMT in cancer are fundamentally dependent on actin cytoskeleton remodeling. Enhanced motility, invasiveness, and poor clinical outcomes are commonly linked to altered expression of ABPs, including cofilin, fascin, profilin, gelsolin, and ezrin, which are often dysregulated in malignancies [8].

Recent research has revealed that miRNAs critically regulate the expression and function of ABPs, thereby altering cytoskeletal dynamics and modulating the behavior of ovarian cancer cells [9]. The miR-200 family impedes EMT and metastasis by targeting fascin-1, an actin-binding protein that enhances cell motility and filopodia formation via the bundling of F-actin filaments. Actin filament turnover also depends on cofilin and gelsolin, which have been demonstrated to be regulated by miR-145 and miR-21, respectively [10,11].

However, publications that specifically summarize these relationships for ovarian cancer are conspicuously lacking, despite mounting data that link miRNA–ABP networks to the advancement of disease. Given that more than 70% of patients with ovarian cancer receive a diagnosis at an advanced stage, when metastases and chemoresistance are the main causes of five-year survival rates falling below 30%, this disparity is particularly serious [12,13]. Therefore, there is an urgent need to comprehend how cytoskeletal remodeling caused by miRNA-regulated ABPs contributes to these clinical difficulties.

Despite the growing association between miRNA-ABP regulatory networks and the advancement of cancer, little is known about the precise processes by which actin-binding proteins support chemoresistance, especially taxol resistance, in ovarian cancer. To clarify how these proteins cause resistance and to find possible therapeutic targets, a thorough synthesis centered on ovarian cancer is required, given the crucial role that ABPs play in regulating cytoskeletal dynamics, cell motility, and drug efflux routes [14]. An enhanced understanding of the disease’s etiology, along with the identification of novel biomarkers and therapeutic targets, relies on comprehensive knowledge of these molecular interactions [15].

This narrative review goes beyond previous descriptions by concentrating on the regulation of actin-binding proteins by miRNAs in ovarian cancer, a regulatory axis that has not yet been thoroughly constructed. The potential of miRNA–ABP networks as biomarkers and therapeutic targets is discussed, along with new evidence that links them to cytoskeletal remodeling, invasion, metastasis, and chemoresistance. By combining clinical consequences with molecular pathways, we hope to offer a current viewpoint that could direct future investigations and intervention tactics.

## 2. Overview of Actin-Binding Proteins in Cancer

### 2.1. Structure and Function of ABPs

Key physiological processes such as intracellular transport, motility, and cell division are enabled by ABPs, which also play a crucial role in regulating the dynamic and adaptable network of actin filaments comprising the cytoskeleton. The actin cytoskeleton is primarily composed of filamentous actin (F-actin) polymers, which are assembled from globular actin (G-actin) monomers. The assembly and disassembly of actin filaments are tightly controlled by various ABPs through their regulation of nucleation, elongation, crosslinking, severing, and capping. In response to external stimuli and intracellular signals, the cytoskeleton undergoes constant remodeling, driven by the coordinated actions of these proteins [16,17].

Cofilin, a small actin-severing protein that binds to ADP-actin filaments and encourages depolymerization, enhances actin turnover, a crucial step for cell motility [18]. Profilin facilitates actin filament elongation by accelerating the exchange of ADP for ATP on G-actin and priming actin monomers for polymerization [19]. The actin-bundling protein fascin arranges parallel F-actin filaments into densely packed bundles, facilitating the formation of filopodia—thin projections located at the leading edge of migrating cells [20]. Another important ABP, gelsolin, performs dual functions: it severs actin filaments and, in a calcium-dependent manner, caps their rapidly growing ends, thereby enabling rapid cytoskeletal remodeling [21]. The Arp2/3 complex, together with other regulatory proteins, facilitates the nucleation of new actin filaments and the formation of branched networks required for lamellipodium development during cell migration [22].

These ABPs work in concert to maintain a precise equilibrium between actin filament assembly and disassembly, thereby enabling cells to dynamically adjust their shape and motility in response to external cues [23]. Although vital for normal cellular function, the deregulation of these mechanisms can substantially impact cell behavior, especially in relation to cancer development and dissemination [24]. Table 1 summarizes key actin-binding proteins, their primary functions in cytoskeletal regulation, and their roles in cancer.

Actin networks regulated by these ABPs in cancer cells exhibit dynamic behavior that has been visually recorded in a number of imaging investigations. Actin filaments can be quickly reorganized into structures like lamellipodia, filopodia, or stress fibers in response to external stimuli like growth factors or modifications in the tumor microenvironment, as seen by photomicrographs from live-cell imaging and fluorescence microscopy. In addition to confirming the molecular functions of ABPs, including cofilin, fascin, and the Arp2/3 complex, these visualizations shed light on how cytoskeletal remodeling supports the invasion and motility of cancer cells [10,25,26,27]. Although this narrative overview does not cover original imaging data, we direct readers to important publications that provide representative depictions of these dynamic processes.

### 2.2. ABPs in Cancer Metastasis

In cancer, the dysregulation of ABPs is frequently associated with enhanced invasiveness, metastatic potential, and resistance to therapy [28]. Since metastasis involves a series of complex, actin-dependent processes—such as the loss of cell–cell adhesion, degradation of the extracellular matrix, and migration through tissues—ABPs are often hijacked by cancer cells to facilitate these events [29]. Fascin-1 is among the most extensively studied actin-binding proteins in the context of cancer. Fascin-1 is often absent or low in epithelial tissues, but it is increased in numerous carcinomas, such as colorectal, breast, and ovarian malignancies. Its overexpression has been associated with poor clinical outcomes, increased invasiveness, and enhanced cell motility. Fascin-1 expression has been linked to advanced stages of ovarian cancer and a lower overall survival rate, indicating that it may be useful as a prognostic biomarker [30].

Cofilin, a critical actin-binding protein, plays a role in mediating the directed migration of cancer cells. Cofilin promotes the formation of lamellipodia and invadopodia, structures that facilitate tissue invasion, by enhancing actin turnover at the leading edge of migrating cells [31]. Increased cofilin activity, reported across multiple cancer types, may intensify chemotherapy resistance. In certain situations, gelsolin can promote apoptosis and act as a tumor suppressor in cancer. To enable tumor cells to withstand separation from the extracellular matrix and withstand apoptosis during intraperitoneal spread, ovarian cancer requires cytoskeleton remodeling and anoikis suppression for metastatic dissemination within the peritoneal cavity. This cytoskeletal rearrangement promotes tumor growth by facilitating cell invasion and movement [32,33]. Furthermore, ezrin, although not a classical ABP, links the actin cytoskeleton to membrane proteins and is critical in metastasis and EMT. It contributes to the generation of protrusive structures required for cell invasion and is commonly overexpressed in metastatic cancers [34].

The altered expression and activity of ABPs are fundamental to the metastatic cascade, encompassing processes from detachment at the primary tumor site to the colonization of distant organs. In addition to indicating the invasive phenotype of cancer cells, their expression profiles identify potential targets for therapies designed to inhibit metastasis [35]. The major ABPs involved in cancer metastasis and their dual roles in normal cellular processes and tumor progression are outlined in Table 2.

## 3. Role of microRNAs in Cancer

### 3.1. Biogenesis and Mechanism of Action

miRNAs represent a class of small, non-coding RNAs which are essential for the post-transcriptional regulation of gene expression [5]. The synthesis of miRNAs begins in the nucleus, where RNA polymerase II transcribes miRNA genes into long primary transcripts known as primary miRNAs (pri-miRNAs), initiating a multi-step biogenesis pathway. These pri-miRNAs have distinctive hairpin shapes and are usually several hundred nucleotides long. The pri-miRNAs are broken down into ~70-nucleotide precursor miRNAs (pre-miRNAs) by the nuclear microprocessor complex, which comprises the RNase III enzyme Drosha and its cofactor DGCR8. Then, in a Ran-GTP-dependent process, Exportin-5 exports the pre-miRNAs to the cytoplasm [36,37,38].

Once in the cytoplasm, the pre-miRNAs are further processed by the RNase III enzyme Dicer, which cuts the loop area of the hairpin to generate a double-stranded RNA duplex [39]. Usually destroyed, this duplex comprises the mature miRNA strand, also known as the guide strand, along with the complementary passenger strand. The mature miRNA is subsequently incorporated into the RNA-induced silencing complex (RISC), which guides it to target mRNAs by base-pairing with complementary sequences, predominantly located within the 3′ UTRs of the transcripts [40].

**Figure 1:**
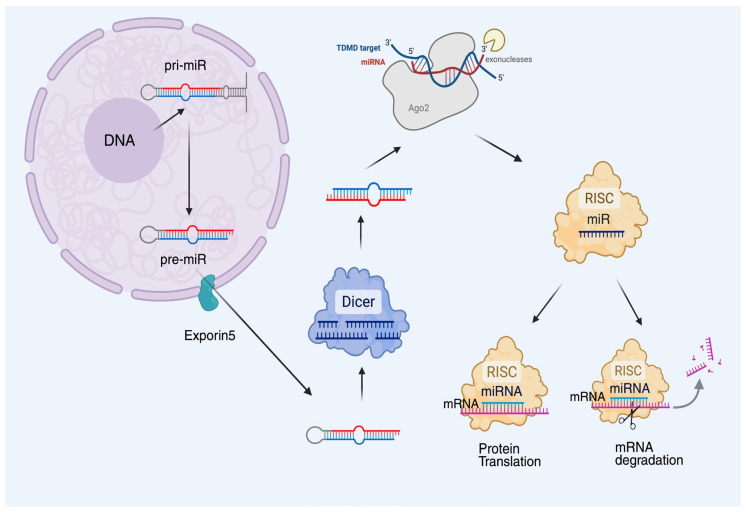
Schematic overview of microRNA-mediated regulation of gene expression. Mature miRNAs are loaded into the RNA-induced silencing complex (RISC), where they guide the complex to complementary sequences in the 3′ untranslated regions (3′ UTRs) of target mRNAs. This interaction results in translational repression and/or mRNA degradation, ultimately leading to reduced protein output. Ago2: Argonaute 2, TDMD: target-directed miRNA degradation.

The outcome is either mRNA destruction or translation inhibition, depending on how complementary the miRNA and its target are [41]. From differentiation to death, and from proliferation to stress reactions, miRNAs coordinate a wide range of cellular activities, including immunological regulation. The complexity and versatility of miRNA-mediated regulation are evidenced by the ability of a single miRNA to target hundreds of distinct mRNAs, as well as the capacity for multiple miRNAs to concurrently regulate a single mRNA (Figure 1) [42].

### 3.2. Oncogenic and Tumor-Suppressive miRNAs

In the context of cancer, miRNAs play dual roles and can function either as oncogenes (oncomiRs) or tumor suppressors, depending on the biological context and the identity of their target genes [43]. The dysregulation of miRNA expression is a common feature in human malignancies and contributes to all hallmarks of cancer, including sustained proliferative signaling, the evasion of growth suppressors, resistance to cell death, the induction of angiogenesis, the activation of invasion and metastasis, and metabolic reprogramming [44,45].

Many miRNAs have been found to have either tumor-suppressive or oncogenic properties in ovarian cancer. By targeting transcriptional repressors of E cadherin, such as ZEB1 and ZEB2, the miR 200 family, including miR 200a, miR 200b, miR 200c, miR 141, and miR 429, is known to inhibit tumor growth, prevent EMT, and decrease the risk of metastasis [46,47]. Conversely, miR-21, one of the most frequently upregulated oncomiRs in various cancers, promotes tumor progression in ovarian cancer by targeting and downregulating tumor suppressor genes such as PTEN and PDCD4, facilitating cell survival, proliferation, and invasion [48].

Other miRNAs that inhibit oncogenes and ABPs involved in cell motility and cytoskeletal reorganization, like let-7, miR-145, and miR-34a, have tumor-suppressive effects [6]. On the other hand, oncomiRs such as miR-182 and miR-221/222 have been linked to improved cell migration and greater resistance to chemotherapy. To further connect cytoskeletal dynamics with oncogenic signaling pathways, many of these miRNAs also play a role in controlling the expression of actin-binding proteins [49].

Overall, aberrant miRNA expression promotes the initiation, progression, and metastasis of ovarian cancer by regulating genes that play key roles in essential biological pathways. miRNAs are also desirable candidates for application as therapeutic agents and diagnostic indicators due to their selectivity for gene targets, stability in bodily fluids, and relatively small size [50,51].

## 4. MiRNA Regulation of ABPs in Ovarian Cancer

MiRNAs are essential for regulating the networks of gene expression that control cellular motility and architecture. The control of ABPs by individual miRNAs is one of the newest topics in cancer biology, especially in tumors like ovarian carcinoma, where cellular invasion and migration are characteristics of the disease’s course. Several studies have begun to delineate the specific miRNA–ABP interactions that influence cytoskeletal dynamics and metastatic behavior in ovarian cancer cells [9,52].

### 4.1. miR-200 Family and Fascin

More research has been conducted on the miR-200 family of miRNAs in ovarian cancer than on any other epithelial malignancy [53]. The well-known tumor-suppressive properties of miR-200a, miR-200b, miR-200c, miR-141, and miR-429 are included, particularly through the inhibition of EMT. Epithelial cells undergo EMT, which results in increased motility, invasiveness, and apoptosis resistance [54]. FSCN1, the gene producing fascin, a well-known actin-bundling protein involved in the development of filopodia and other protrusive structures that aid in cell migration, is one of the main downstream targets of the miR-200 family. The miR 200 family targets FSCN1, which results in the downregulation of fascin expression and limits the creation of invasive cytoskeletal structures, decreases cell motility, and suppresses the potential for metastasis [55,56].

### 4.2. miR-21 and Gelsolin

MiR-21 is among the most commonly upregulated oncomiRs in multiple cancers, including ovarian cancer. It promotes cancer by targeting and inhibiting several tumor suppressor genes involved in apoptosis, DNA repair, and cytoskeletal control. It targets gelsolin, an actin-severing and -capping protein that controls actin filament turnover in a calcium-dependent manner [57,58].

Gelsolin plays a multifaceted role in cancer, operating as a tumor suppressor and promoter depending on the situation. In ovarian cancer, miR-21-mediated gelsolin downregulation disturbs normal actin dynamics, which may contribute to increased cell survival, resistance to anoikis, and decreased sensitivity to apoptotic signals [59,60]. A reduction in gelsolin function can help to stabilize actin structures that facilitate cellular adhesion and migration, allowing cancer cells to spread more easily. Thus, miR-21 indirectly increases invasive behavior in ovarian cancer by modulating the cytoskeletal architecture [61].

### 4.3. Other miRNA-ABP Interactions

Recent research has uncovered other microRNAs that modulate actin-binding proteins, influencing key cellular activities including cytoskeletal rearrangement, migration, and invasion, thereby extending the known miRNA-ABP regulatory network in ovarian cancer [62]. MiR-145 exhibits tumor-suppressive effects by targeting cofilin-1, a vital protein involved in actin filament depolymerization. Cofilin-1 regulates actin filament turnover and is required for cancer cell migration via cytoskeletal remodeling. In ovarian cancer, miR-145 downregulation increases cofilin-1 activity, promoting more migratory and invasive behavior. The re-expression of miR-145 reduces cofilin-1 levels, promotes actin filament stability, and inhibits the formation of metastatic protrusions, underscoring its promise as a therapeutic strategy in ovarian cancer [63,64].

MiR-138 serves an important function by targeting ezrin, a cytoskeletal linker protein that connects the actin cytoskeleton to the plasma membrane. The overexpression of ezrin has been linked to higher metastatic potential and poor clinical outcomes in patients with ovarian cancer [34,65]. Through direct inhibition of ezrin, miR-138 remodels the cytoskeleton, reducing cancer cells’ capacity to generate migratory and invasive structures like filopodia and lamellipodia [10]. As a result, the miR-138/ezrin regulation axis is an important route via which miRNAs can influence the invasive aggressiveness of ovarian cancer cells. These findings highlight the larger regulatory network through which miRNAs affect actin-binding proteins and cytoskeletal dynamics (Table 3) [66,67].

## 5. Clinical Implications

The dysregulation of miRNAs in ovarian cancer carries significant clinical implications, especially regarding diagnosis, prognosis, and therapeutic strategies. MiRNAs that regulate ABPs are emerging as attractive options in the therapeutic treatment of ovarian cancer due to their stability in biological fluids and ability to reflect underlying tumor biology [68,69].

### 5.1. Diagnostic and Prognostic Potential

Several miRNAs involved in ABP control have demonstrated potential as non-invasive biomarkers. Notably, miR-200 and miR-21 are consistently identified in both serum and tumor tissues of individuals with ovarian cancer [70]. MiR-200, which is essential for retaining epithelial features and controlling EMT, is frequently reduced in advanced ovarian cancer. Conversely, miR-21, an oncogenic miRNA that targets tumor suppressor genes and modulates actin cytoskeletal dynamics, is often upregulated and has been associated with unfavorable clinical outcomes [58,71]. Serum identification of these miRNAs aids in early diagnosis and can discriminate between benign and malignant ovarian cancers. Furthermore, continuous monitoring of circulating miRNA levels may provide a minimally invasive approach to assess disease progression and therapeutic response [72,73].

### 5.2. Therapeutic Targeting

In addition to their diagnostic utility, miRNAs are promising therapeutic agents in ovarian cancer. Restoring tumor-suppressive miRNAs like miR-145 and miR-138 or blocking oncogenic miRNAs like miR-21 can alter actin-binding proteins like cofilin 1 and ezrin, which will alter cytoskeletal dynamics and lessen invasiveness [48,74,75]. Importantly, significant therapeutic benefits have been shown by in vivo modification of the miRNA–ABP pathways. For instance, in orthotopic ovarian cancer xenografts, systemic administration of an miR-506 mimic utilizing DOPC nanoparticles decreased tumor burden by more than 90% when paired with chemotherapy, mostly due to RAD51 downregulation and improved chemosensitivity [76]. Similarly, in CD44^+^/CD117^+^ xenograft models, lentiviral or agomiR-mediated restoration of miR-200c postponed tumor development, inhibited metastasis, and enhanced survival, especially when combined with cisplatin and paclitaxel (*p* < 0.01) [77]. By focusing on DNA repair pathways as well as the cytoskeletal machinery that underlies tumor progression, these findings highlight the potential of miRNA-based therapies to prevent metastasis and overcome chemoresistance.

Personalized therapeutic approaches are also made possible by recent developments in miRNA profiling, which make it possible to choose particular miRNA interventions (like miR-506 or miR-200c) as effective supplements to standard treatment plans for destroying the molecular causes of ovarian tumor growth and metastasis [78,79].

## 6. Challenges and Future Perspectives

Despite growing interest in exploiting miRNA–ABP interactions for therapeutic and diagnostic applications in ovarian cancer, several critical challenges must be addressed before these strategies can be translated into clinical practice [80].

The specificity of miRNA function is one significant obstacle. Since individual miRNAs frequently have several mRNA targets, therapeutic manipulation of these molecules may have broad and unexpected consequences. This lack of target exclusivity complicates efforts to precisely modulate specific ABP-related pathways without disrupting other essential cellular processes. Mitigating off-target effects will require an in-depth understanding of complex regulatory networks and careful consideration of context-specific influences [74].

Delivery efficiency is still a major problem. The successful clinical translation of miRNA-based therapies depends on the development of efficient, targeted, and biocompatible delivery mechanisms [74,81]. miRNA mimics and inhibitors must be delivered to tumor cells in sufficient quantities while avoiding degradation in circulation, immune activation, or accumulation in non-target tissues [82]. Viral vectors and nanoparticle-based strategies show considerable promise; however, ongoing refinement is essential to secure safety, maintain stability, and achieve tumor-specific delivery [80,83].

In preclinical ovarian cancer models, for instance, lipid-based nanoparticles and exosome-mediated delivery have been investigated and shown to improve miRNA stability and tumor targeting while lowering systemic toxicity [84]. Although they have also demonstrated potential, viral vectors such as adeno-associated viruses (AAVs) give rise to worries over insertional mutagenesis and immunological responses. In order to balance delivery effectiveness and safety, these methods highlight the necessity for ongoing adjustment [85]. Off-target effects are still a serious worry because they can have detrimental cellular effects through inadvertent interactions with non-target mRNAs. This emphasizes the significance of accurate delivery and thorough preclinical testing.

Furthermore, validation of miRNA–ABP interactions in vivo remains limited. Despite the valuable molecular insights gained from in vitro research, comprehensive in vivo models and clinical trials are essential to establish the therapeutic relevance of these pathways [86]. Organoids, 3D culture techniques, and patient-derived xenograft models represent valuable tools for assessing miRNA activity in biologically relevant environments [87]. Furthermore, larger patient cohorts and longitudinal studies are necessary to evaluate the prognostic value of circulating miRNAs and their consistency across diverse disease stages and subtypes [88].

The intricate control of tumor cell motility is further clarified by recent research on actin-based migration in cancer cells. For instance, Laura M. Machesky’s group’s paper (2025) used sophisticated microscopy and migration assays to show how the β3 subunit controls glioma cell movement by remodeling the actin cytoskeleton, regardless of its ion channel activity [89]. The fundamental ideas of actin-driven migration apply to ovarian cancer, where cytoskeletal dynamics also control invasive behavior, even though the study concentrated on glioma cells. The complex regulation of cell motility in ovarian cancer is highlighted by integrating these findings with our analysis of the microRNA-mediated regulation of actin-binding proteins. This analysis identifies possible shared pathways and molecular targets that may be used therapeutically to prevent the migration and metastasis of cancer cells.

Future investigations should prioritize the integration of multi-omics methodologies—including transcriptomics, proteomics, and epigenomics—to uncover key regulatory nodes within the miRNA–ABP axis. Such integrative analyses could uncover master regulators of cytoskeletal remodeling and help prioritize miRNA targets for therapeutic development [90].

Furthermore, employing artificial intelligence and systems biology approaches to model miRNA–target networks may accelerate the identification of functionally relevant interactions. Validating these results in patient-derived models will ultimately be essential to bridging the gap between bench and bedside [91].

In summary, although therapeutic targeting of miRNA–ABP interactions in ovarian cancer presents considerable promise, advancing their clinical application requires a more comprehensive understanding of underlying biological mechanisms, enhanced delivery systems, and thorough clinical validation.

## 7. Conclusions

ABPs and microRNAs interact intricately, and this interaction is a key regulatory axis in the development and spread of ovarian cancer. MiRNAs regulate key tumorigenic processes such as cell migration, invasion, epithelial-to-mesenchymal transition, and metastasis by modulating the expression and function of vital cytoskeletal regulators [52]. The dual roles of miRNAs as tumor suppressors and oncogenes are increasingly recognized, highlighting their complexity and therapeutic potential, which vary according to target specificity and cellular context [6].

One intriguing approach to improving ovarian cancer early detection, prognosis, and treatment is to target the miRNA–ABP axis. Preclinical studies suggest that, while circulating miRNAs such as miR-200, miR-21, and miR-145 have demonstrated relevance as non-invasive biomarkers, therapeutic approaches aimed at restoring or suppressing specific miRNAs may modulate ABP function and restrict metastatic progression. Critical challenges such as achieving target selectivity, improving delivery efficiency, and obtaining robust in vivo validation must be addressed to translate these findings into effective therapeutic applications [12].

Future research should concentrate on identifying the molecular mechanisms underlying miRNA–ABP interactions, defining regulatory networks using multi-omics data, and evaluating treatment targets in clinically relevant models. In conclusion, enhanced insight into the regulatory role of miRNAs in actin-binding proteins may significantly improve our understanding of ovarian cancer biology and enable the development of novel diagnostic and therapeutic strategies. Translating this molecular insight into clinical practice holds substantial promise for improving outcomes in patients with ovarian cancer [92,93].

## Figures and Tables

**Table 1:** This table highlights the main ABPs, their physiological roles, and their specific contributions to cytoskeleton remodeling.

Protein/Complex	Primary Function in Normal Cells	Role in Actin Cytoskeleton Dynamics
Cofilin	Actin-severing protein; binds ADP-actin filaments	Enhances depolymerization and actin turnover, supporting cell motility
Profilin	Facilitates nucleotide exchange on G-actin monomers	Accelerates ADP-to-ATP exchange; primes actin monomers for polymerization
Fascin	Actin-bundling protein	Arranges parallel F-actin filaments into dense bundles; forms filopodia
Gelsolin	Severing and capping protein	Severs filaments; calcium-dependent capping of filament ends, enabling remodeling
Arp2/3 complex	Actin nucleation complex	Initiates nucleation of new filaments; forms branched networks for lamellipodia

**Table 2:** This table captures how each ABP contributes to metastasis and its clinical significance.

Protein	Role in Cancer Metastasis	Clinical/Functional Implications
Cofilin	Promotes lamellipodia and invadopodia formation, enhancing cancer cell migration and tissue invasion	Increased activity linked to chemotherapy resistance and enhanced metastatic potential
Fascin-1	Overexpressed in many carcinomas (colorectal, breast, ovarian); promotes cell motility and invasiveness	Associated with poor prognosis, advanced cancer stages, and lower overall survival
Gelsolin	Dual role: can promote apoptosis (tumor suppressor) or aid tumor progression by remodeling cytoskeleton and inhibiting anoikis	Context-dependent function, impacting tumor progression or suppression
Ezrin	Links actin cytoskeleton to membrane proteins; facilitates protrusions for invasion	Overexpressed in metastatic cancers; critical for metastasis and EMT

**Table 3:** This table summarizes the regulatory interactions between specific miRNAs and ABPs, highlighting their functional effects on cancer-related cellular processes and their clinical significance.

miRNA	Target ABP	Functional Effect	Clinical Relevance
miR-200	Fascin	↓ Invasion, ↓ EMT	Prognostic marker
miR-21	Gelsolin	↑ Proliferation, ↓ Apoptosis	Therapeutic resistance
miR-145	Cofilin	↓ Migration	Tumor suppressor role
miR-138	Ezrin	↓ Invasion	EMT suppression

↑ means increase of a functional effect, ↓ means decrease of a functional effect.

## Data Availability

No new data were created or analyzed in this study. Data sharing is not applicable to this article.
